# Identifying amyloid pathology–related cerebrospinal fluid biomarkers for Alzheimer's disease in a multicohort study

**DOI:** 10.1016/j.dadm.2015.06.008

**Published:** 2015-08-01

**Authors:** Yuk Yee Leung, Jon B. Toledo, Alexey Nefedov, Robi Polikar, Nandini Raghavan, Sharon X. Xie, Michael Farnum, Tim Schultz, Young Baek, Vivianna M. Van Deerlin, William T. Hu, David M. Holtzman, Anne M. Fagan, Richard J. Perrin, Murray Grossman, Holly D. Soares, Mitchel A. Kling, Matthew Mailman, Steven E. Arnold, Vaibhav A. Narayan, Virginia M-Y. Lee, Leslie M. Shaw, David Baker, Gayle M. Wittenberg, John Q. Trojanowski, Li-San Wang

**Affiliations:** a Department of Pathology & Laboratory Medicine, Institute on Aging, Institute for Biomedical Informatics, Perelman School of Medicine at the University of Pennsylvania, Philadelphia, PA, USA; b Department of Pathology & Laboratory Medicine, Institute on Aging, Center for Neurodegenerative Disease Research, Philadelphia, PA, USA; c Department of Pathology & Laboratory Medicine, Perelman School of Medicine at the University of Pennsylvania, Philadelphia, PA, USA; d Department of Electrical and Computer Engineering, Rowan University, Glassboro, NJ, USA; e Department of Quantitative Science, Janssen Research & Development, LLC, Titusville, NJ, USA; f Department of Biostatistics and Epidemiology, Perelman School of Medicine at the University of Pennsylvania, Philadelphia, PA, USA; g Department of Neuroscience, Janssen Research & Development, LLC, Titusville, NJ, USA; h Department of Neurology, Emory University School of Medicine, Atlanta, GA, USA; i Department of Neurology, Knight Alzheimer's Disease Research Center, Hope Center for Neurodegenerative Disorders, Washington University, St Louis, MO, USA; j Department of Neurology, Knight Alzheimer's Disease Research Center, Hope Center for Neurodegenerative Disorders, Washington University, St Louis, MO, USA; k Department of Pathology and Immunology, Division of Neuropathology, Knight Alzheimer Disease Research Center, Hope Center for Neurological Disorders, Washington University, St Louis, MO, USA; l Department of Neurology, Perelman School of Medicine at the University of Pennsylvania, Philadelphia, PA, USA; m Clinical Biomarkers, Bristol-Meyer Squibb, Hopewell, NJ, USA; n Behavioral Health Service, Philadelphia VA Medical Center, Department of Psychiatry, University of Pennsylvania School of Medicine, Philadelphia, PA, USA; o Department of Neurology, Department of Psychiatry, Perelman School of Medicine at the University of Pennsylvania, Philadelphia, PA, USA

**Keywords:** Cerebrospinal fluid, Biomarkers, Alzheimer's disease, Cognitive impairment, Amyloid beta, Dementia

## Abstract

**Introduction:**

The dynamic range of cerebrospinal fluid (CSF) amyloid β (Aβ_1–42_) measurement does not parallel to cognitive changes in Alzheimer's disease (AD) and cognitively normal (CN) subjects across different studies. Therefore, identifying novel proteins to characterize symptomatic AD samples is important.

**Methods:**

Proteins were profiled using a multianalyte platform by Rules Based Medicine (MAP-RBM). Due to underlying heterogeneity and unbalanced sample size, we combined subjects (344 AD and 325 CN) from three cohorts: Alzheimer's Disease Neuroimaging Initiative, Penn Center for Neurodegenerative Disease Research of the University of Pennsylvania, and Knight Alzheimer's Disease Research Center at Washington University in St. Louis. We focused on samples whose cognitive and amyloid status was consistent. We performed linear regression (accounted for age, gender, number of apolipoprotein E (*APOE*) e4 alleles, and cohort variable) to identify amyloid-related proteins for symptomatic AD subjects in this largest ever CSF–based MAP-RBM study. ANOVA and Tukey's test were used to evaluate if these proteins were related to cognitive impairment changes as measured by mini-mental state examination (MMSE).

**Results:**

Seven proteins were significantly associated with Aβ_1–42_ levels in the combined cohort (false discovery rate adjusted *P* < .05), of which lipoprotein a (Lp(a)), prolactin (PRL), resistin, and vascular endothelial growth factor (VEGF) have consistent direction of associations across every individual cohort. VEGF was strongly associated with MMSE scores, followed by pancreatic polypeptide and immunoglobulin A (IgA), suggesting they may be related to staging of AD.

**Discussion:**

Lp(a), PRL, IgA, and tissue factor/thromboplastin have never been reported for AD diagnosis in previous individual CSF–based MAP-RBM studies. Although some of our reported analytes are related to AD pathophysiology, other's roles in symptomatic AD samples worth further explorations.

## 1 Introduction

Alzheimer's disease (AD) is pathologically characterized by the presence of extracellular amyloid plaques (APs) and intracellular hyperphosphorylated tau neurofibrillary tangles, which are known to be correlated with cerebrospinal fluid (CSF) levels of amyloid β (Aβ_1–42_), total tau (t-tau), and phosphorylated tau (p-tau_181_) [1], [2]. The measurements of these proteins in the CSF using enzyme-linked immunosorbent assay (ELISA) and xMAP technology were able to distinguish most AD and cognitively normal (CN) subjects [3], [4]. These CSF biomarkers are included in the revised version of the commonly used diagnosis criteria Institute of Neurological and Communicative Disorders and Stroke (NINCDS) and the Alzheimer's Disease and Related Disorders Association (ADRDA) in 2011 for supporting clinical diagnoses [5]. Analyses of CSF Aβ_1–42_, t-tau, and p-tau_181_ in a meta-analysis study (combining 11 different studies) were shown to accurately classify AD patients (area under the curve, 0.86) [6]. Nevertheless, CSF Aβ_1–42_ reaches pathologic values and then plateau during the preclinical phase of the disease, when subjects still have normal cognition, and therefore show low correlation with cognitive symptoms [7]. Although CSF t-tau levels show a better correlation with cognition, there is a need for additional CSF biomarkers that track cognitive changes closely. Due to the heterogeneity of the disease populations, it is critical to validate identified biomarker candidates across different cohorts.

Recent studies have been conducted to identify and characterize other potential CSF biomarkers, as reviewed by Fagan and Perrin [8]. These include visinin-like protein-1 and chitinase 3-like 1 (cartilage glycoprotein-39; YKL-40) for which follow-up studies explored their roles in different disease populations [9], [10], [11]. However, disappointingly, most of the other candidate biomarkers have not been replicated to date. Comparing to Aβ_1–42_ and t-tau, they possibly participate in different time frames in the AD spectrum [12], [13]. Therefore, by combining cohorts comprised subjects with different levels of cognitive deficits, we postulate that the candidate biomarkers may better explain the disease progression in a heterogeneous population defined by cognitive measures such as mini-mental state examination (MMSE) as opposed to more global clinical status (AD vs. CN).

Multiplex methods can identify CSF biomarkers altered in AD and have utility as potential diagnostic and disease staging tools, and for nominating novel drug targets and tracking treatment responses for investigational interventions. Hu et al. [14] previously conducted a study on subjects from the University of Pennsylvania (UPenn) using the Human DiscoveryMAP panel from Rules Based Medicine (MAP-RBM), where they identified CSF biomarkers (including thirteen analytes from the MAP-RBM) for distinguishing pathologically confirmed AD from CN subjects. Another study involved subjects recruited at Knight Alzheimer's Disease Research Center at Washington University in St. Louis (WUSTL), in which biomarkers were identified to distinguish very mild and mild AD from CN subjects [15]. In a recent study on subjects from Alzheimer's Disease Neuroimaging Initiative (ADNI) [16], Mattsson et al. focused on 46 healthy control subjects and showed that some proteins from the MAP-RBM panel can predict future Aβ_1–42_ reduction in subjects with normal baseline Aβ_1–42,_ suggesting they can pathologically predict future development of the brain APs at the earliest stages of AD, before their widespread development.

Although all studies described used the same MAP-RBM panel, results could not be directly comparable for two reasons. First, each study compared a specific stage of AD samples (pathologically confirmed AD in UPenn, mild AD in WUSTL) to CN. Second, their preprocessing steps were different—only ADNI data were log-transformed. Driven by this, we believe it would be of great value to create a new cohort by combining all these MAP-RBM data from all cohorts (ADNI—a clinical trial type cohort, UPenn—a tertiary care memory center, and WUSTL—a community-dwelling research cohort). These data thus contain subjects of different levels of cognitive deficits. Given such a heterogeneous population, the novelty in our study lies in identifying candidate biomarkers that may better explain the disease progression instead of diagnosis. The purpose of our study was twofold: (1) to identify MAP-RBM analytes suggestive of the presence or absence of amyloid pathology quantified by CSF Aβ_1–42_ levels (Aβ_1–42_ cutoff defined by Shaw et al. [17] regardless of clinical diagnoses) and (2) study how these biomarkers correlate with cognitive performance. Despite our different study focus thus limiting our choice of subjects from each individual studies [14], [15], [16], we have the largest sample size so far for MAP-RBM study in AD. Also our data have more similar numbers of symptomatic cases and CN controls as compared with individual studies. To summarize our multicenter study, we first applied same preprocessing steps including imputation for low values, excluding outliers and normalization for MAP-RBM on all cohorts. Because there are cohort-specific demographics, we merged all cohorts together, adjusted for age, gender, and number of *APOE* *ε*4 alleles, and used the cohort indicator as an additional covariate to control for batch effects. We first calculated the correlation of these analytes with Aβ_1–42_ levels, then evaluated their utility to differentiate subjects with different cognitive problems.

## 2 Materials and methods

### 2.1 Participants, biomarker collection, and analysis tools

Part of the data used in the preparation of this article were obtained from the ADNI database (adni.loni.ucla.edu). The ADNI was launched in 2003 by the National Institute on Aging, the National Institute of Biomedical Imaging and Bioengineering, the Food and Drug Administration, private pharmaceutical companies and nonprofit organizations, as a $60 million, 5-year public-private partnership. The primary goal of ADNI has been to test whether serial magnetic resonance imaging, positron emission tomography, other biological markers, and clinical and neuropsychological assessment can be combined to measure the progression of mild cognitive impairment (MCI) and early AD. Determination of sensitive and specific markers of very early AD progression is intended to aid researchers and clinicians to develop new treatments and monitor their effectiveness, as well as lessen the time and cost of clinical trials.

In ADNI, baseline CSF samples were obtained in the morning after an overnight fasting and processed as previously described [17], [18], [19]. In brief, lumbar puncture (LP) was performed via aspiration with a 20- or 24-gauge spinal needle as described in the ADNI procedures manual (http://www.adni-info.org/). CSF was collected into polypropylene collection tubes or syringes provided to each site, then transferred into polypropylene transfer tubes without any centrifugation step, followed by freezing on dry ice within 1 hour after collection, and shipped overnight to the ADNI Biomarker Core laboratory at the University of Pennsylvania Medical Center on dry ice. Aliquots (0.5 mL) were prepared from these samples after thawing (1 hour) at room temperature and gentle mixing. The aliquots were stored in bar code–labeled polypropylene vials at −80°C.

Patients and control subjects were recruited and longitudinally followed at UPenn in specialty services dedicated to the evaluation and management of neurodegenerative diseases [14]. All protocols were approved by the Penn Institutional Review Board. Subjects were evaluated at the time of CSF collection, following the similar standard operating procedures as those in ADNI. Biofluid samples were collected up to 3:00 PM during working hours after at least a 4-hour fast. Similarly, LP was performed with a 20- or 24-gauge spinal needle, and CSF was collected via gravity drip or suction method using clear polypropylene tubes and aliquoted into 0.5 mL in 1.5-mL cryogenic tubes after collection without a centrifugation step. Aliquoted samples were sent in sealed containers on dry ice for storage in −80°C in freezers specifically dedicated to banking human biofluid samples.

At WUSTL, participants were volunteers enrolled in longitudinal studies of healthy aging and dementia at the Knight Alzheimer's Disease Research Center [20]. The presence or absence of dementia (and, when present, its severity) was operationalized with the clinical dementia rating (CDR) in accordance with standard protocols and criteria [21]. A CDR of 0 indicates cognitive normality, whereas CDRs of 0.5, 1, 2, and 3 are indicative of very mild, mild, moderate, and severe dementia, respectively. For individuals who are CDR >0, the diagnosis of symptomatic AD is based on NINCDS-ADRDA criteria [22]. Volumes of 25–30 mL of CSF were collected by LP via gravity drip at 8:00 AM after overnight fasting in polypropylene tubes as previously described [23]. Samples were gently inverted to avoid gradient effects, briefly centrifuged at low speed to pellet any cellular elements, and aliquoted (500 μL) into polypropylene tubes before freezing at −84° C. For all biomarker measures, samples were continuously kept on ice, and assays were performed on sample aliquots after a single thaw after initial freezing.

After the samples were collected, they were shipped to Rules Based Medicine (Austin, TX) and evaluated using the MAP-RBM platform, a quantitative multiplexed immunoassay based on Luminex xMAP technology [24], [25]. For ADNI and UPenn cohorts, Aβ_1–42_ levels were measured using xMAP Luminex platform (Luminex Corp, Austin, TX) with Fujirebio (formerly Innogenetics, Ghent, Belgium) immunoassay bead-based kits (INNO-BIA AlzBio3) [17]. According to the manufacturer, AlzBio3 intra-assay variability is <4%, inter-assay variability is <10% [26], and lower limit of quantification is 20–50 pg/mL for Aβ_1–42_ [27]. For the WUSTL cohort, samples were measured using quantitative ELISA with Fujirebio (formerly Innogenetics) immunoassay plate-based kits (INNOTEST) [15]. Because Aβ_1–42_ proteins were not measured using the same assay across the cohorts, and the assays are known to provide different absolute levels (although positively correlated) [3], we used the following formulas to convert WUSTL ELISA levels to their corresponding Luminex estimates: Luminex Aβ_1–42_ = 83.72 + 0.15 × ELISA Aβ_1–42_.

*APOE* genotyping was done similarly across cohorts using DNA from EDTA blood samples: TaqMan allelic discrimination assays were used for nucleotides 334 T/C (rs 429358) and 472 C/T (rs 7412) using a real-time thermocycler (ABI 7500 or 7900; Life Technologies), as previously described [28], [29].

MAP-RBM data from 160 ADNI subjects, 275 UPenn subjects, and 267 WUSTL subjects were available (Supplementary Table 1) [14], [15], [16]. We removed subjects whose Aβ_1–42_ levels disagree with their clinical diagnoses, e.g. AD subjects with Aβ_1–42_ levels (Aβ_1–42_> 192 pg/mL) similar to CN (5, 25, and 3 in ADNI, UPenn, and WUSTL) and normal subjects with low Aβ_1–42_ levels (Aβ_1–42_ <192 pg/mL) typical for AD (33, 3, and 135 in ADNI, UPenn, and WUSTL). This resulted in 122 ADNI subjects, 247 UPenn subjects, and 129 WUSTL subjects (Table 1). The demographics of these cohorts are different as follows: (1) UPenn contains more AD than controls, and the subjects are younger. (2) Gender ratio of ADNI is different from the others. (3) AD subjects in UPenn and WUSTL possess a wider range of cognitive deficit problems.

To study different levels of cognitive impairments, we used MMSE nearest to the time of LP to divide subjects into five stages/groups of cognitive impairment [30]: 29–30 (CN), 26–28 (questionable), 21–25 (mild), 11–20 (moderate), and 0–10 (severe dementia).

### 2.2 Preprocessing MAP-RBM data

As each analyte was analyzed by a specific immunoassay in MAP-RBM and therefore may follow different statistical distributions, special preprocessing steps are required before the analysis. In earlier studies, we identified various ways of preprocessing the MAP-RBM data [14], [15], [16]. To compare the data across three cohorts systematically, we standardized the preprocessing steps as follows:

1. Processing of analytes with missing and low values: Analytes with 10% or more missing or low (“LOW”, defined by the original file obtained from MAP-RBM) values were excluded. For the remaining analytes, entries with “LOW” values were imputed using a value of half of the least detectable dose (LDD) value. The LDD represents the concentration of the analyte that produces a signal above the background level with 99% confidence, which is considered as the most reliable smallest measurement for the protein assays used.
2. Exclusion of outliers: We excluded outliers that were outside five standard deviations from the overall mean.
3. Logarithmic transformation: In ADNI, all analytes not normally distributed were log-10 transformed. In UPenn and WUSTL, a two-step approach was performed. If analytes were also present in ADNI, they were transformed in the same way as in ADNI. If not, they were log-10 transformed if they had a right-skewed distribution. Transforming each analyte to the same distribution across cohorts was essential to ensure proper comparison of these analytes across different cohorts.

A main objective of this study was to identify robust signals that are supported by multiple cohorts despite the heterogeneity of samples. After quality control, 52 analytes were still available across all three cohorts and were retained for analysis (Supplementary Table 2).

### 2.3 Statistical analysis

All analyses were performed using R version 2.14.1 (R Foundation for Statistical Computing). Aβ_1–42_ was not on the MAP-RBM panel and was log-10 transformed such that normality assumptions are satisfied. Univariate analyses of these analytes were tested using linear regression models, adjusting for age at LP, gender, and the number of *APOE* ε4 alleles. In the combined analysis, we used the cohort indicator as an additional covariate to control for batch effects. Analysis of covariance (ANOVA) was conducted on MMSE scores between groups, and Tukey's test was used to assess the statistical significance [31]. Effect sizes were calculated using Cohen's d [32]. All statistical tests were two sided.

## 3 Results

We analyzed MAP-RBM data from the three cohorts to find analytes that were associated with CSF Aβ_1–42_ levels in individuals whose cognitive status was consistent with their amyloid status as defined by CSF Aβ_1–42_ levels (asymptomatic/high Aβ_1–42_ vs. symptomatic/low Aβ_1–42_). First, levels for each analyte were adjusted for age, gender, and number of *APOE* ε4 alleles using ANCOVA (Supplementary Table 3). We observed that the number of *APOE* ε4 alleles had the largest effects on the MAP-RBM analytes in WUSTL, followed by UPenn. The effect of age was significant only in WUSTL. The effect of gender was not significant at all. Given the cohort-specific differences, we included all these covariates in our subsequent models. We performed regression analysis on three cohorts separately, then analyzed the combined data set as described.

### 3.1 MAP-RBM analytes correlated with CSF Aβ_1–42_ levels in individual and combined cohorts

We first performed linear regression to find which MAP-RBM analytes correlated with CSF Aβ_1–42_ levels. Analytes with false discovery rate (FDR) adjusted *P* < .05 (bold text) in at least one cohort are summarized in Table 2 (effect sizes in parenthesis). Complete results are in Supplementary Table 4. We observed that vascular endothelial growth factor (VEGF) and fatty acid binding protein (FABP) were the most significantly associated analytes in three of four analyses, followed by resistin (RETN), which was identified in UPenn and the combined cohort. VEGF, RETN, prolactin (PRL), and lipoprotein a (Lp(a)) have consistent direction of associations across cohorts, as indicated by the sign (positive or negative) of effect sizes, although associations of PRL and Lp(a) were only significant when the three cohorts were combined.

**Fig. 1:**
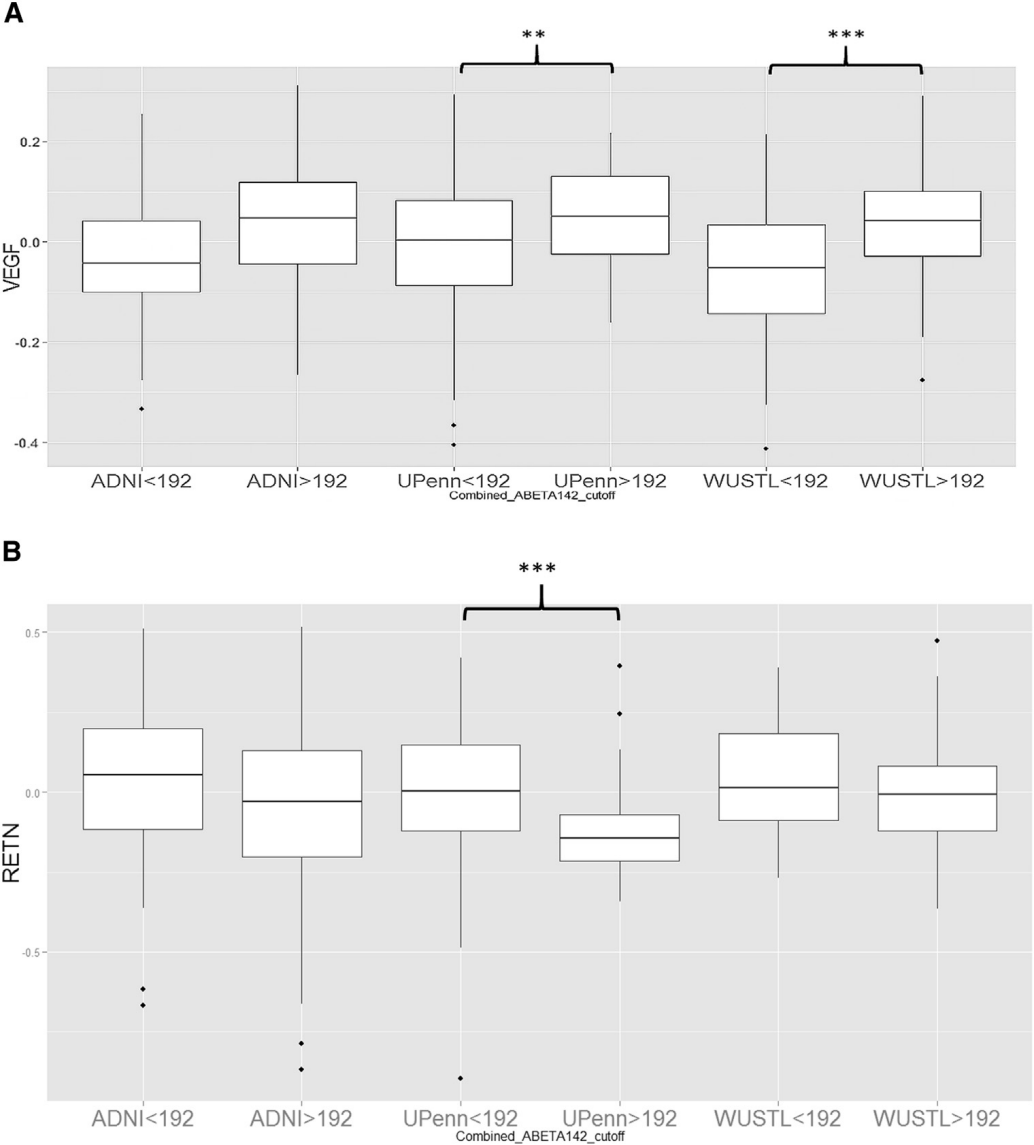
Distributions of MAP-RBM analytes that were found to be mostly associated with CSF Aβ_1–42_ levels from Table 2: (A) VEGF and (B) RETN. Significant codes: **P < .01, ***P < .001. Abbreviations: MAP-RBM, multianalyte platform by Rules Based Medicine; CSF, cerebrospinal fluid; Aβ_1–42_, amyloid β; VEGF, vascular endothelial growth factor; RETN, resistin; ADNI, Alzheimer's Disease Neuroimaging Initiative; UPenn, University of Pennsylvania; WUSTL, Washington University in St. Louis.

The distributions of CSF VEGF and RETN levels are shown in Fig. 1. We observed the direction of the changes associated with diagnosis was consistent across all cohorts, suggesting their robustness. The association between VEGF and CSF Aβ_1–42_ levels was stronger in the symptomatic versus nonsymptomatic group, yet the effect was opposite for that of RETN (results not shown). Boxplots of other candidate MAP-RBM analytes, FABP, CD40 antigen (CD40A), PRL, Lp(a) and hepatocyte growth factor (HGF), are in Supplementary Fig. 1.

### 3.2 Correlation between top MAP-RBM analytes and severity of cognitive impairment

We examined if MAP-RBM analytes were associated levels of cognitive impairment. We focused on subjects that had low Aβ_1–42_ levels (Aβ_1–42_ <192 pg/mL) and MMSE ≤28 and divided them into four groups by MMSE scores (groups “GP26–28” of MMSE = 26–28, “GP21–25” of MMSE = 21–25, “GP11–20” of MMSE = 11–20, and “GP0–10” of MMSE = 0–10). We performed ANOVA to identify analytes that differ among groups. Table 3 summarizes the analytes of which their FDR adjusted *P* values were <.05 in the combined cohort. Three new analytes not found in Table 2 were pancreatic polypeptide (PPP), immunoglobulin A (IgA) and tissue factor/thromboplastin (TF).

**Fig. 2:**
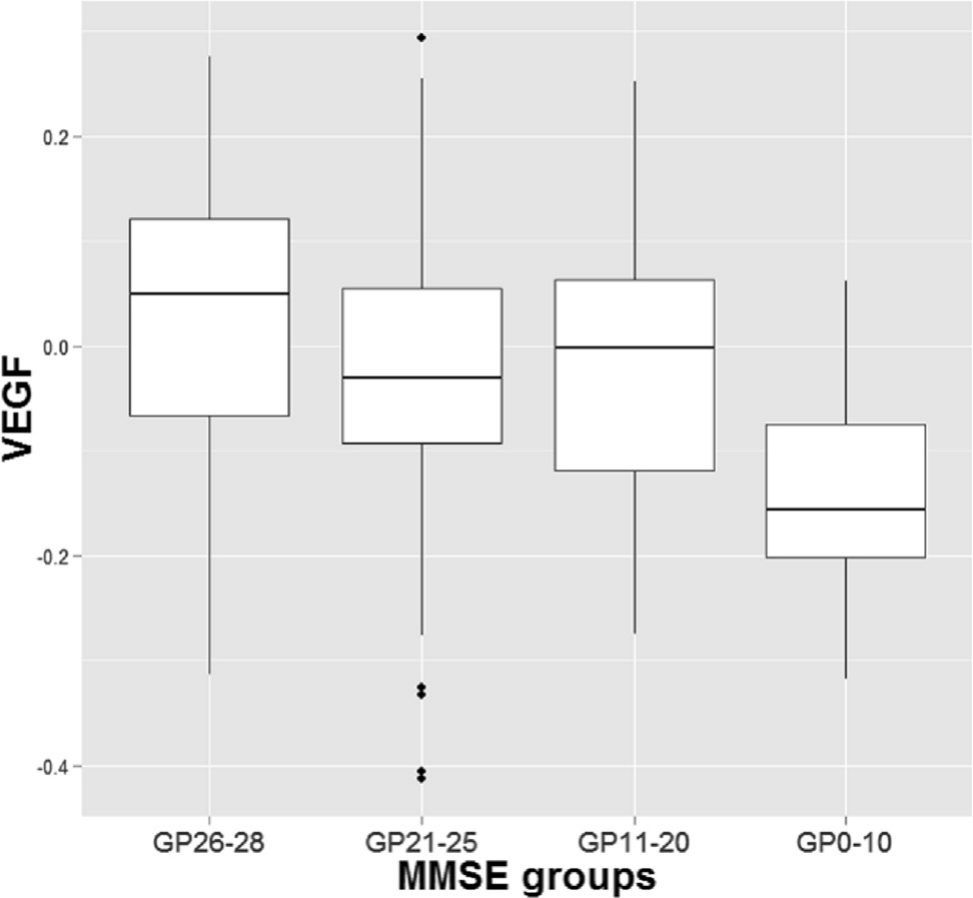
Distributions of VEGF across MMSE groups in the combined cohort. Groups “GP26–28”: MMSE = 26–28; “GP21–25”: MMSE = 21–25; “GP11–20”: MMSE = 11–20; and “GP0–10”: MMSE = 0–10. Abbreviations: VEGF, vascular endothelial growth factor; MMSE, mini-mental state examination.

The distribution of VEGF in combined cohort is shown in Fig. 2 and others in Supplementary Fig. 2. VEGF showed significant differences across certain MMSE groups in UPenn and the combined cohort. As cognitive impairment became worse, CSF level of VEGF decreased. To determine if the differences across MMSE groups were statistically significant, Tukey's test was performed on the combined cohort. At the 95% confidence interval, the group difference between “GP11–20” and “GP0–10” was the most significant (*P* = .014). Other analytes having significant group differences include (1) PPP, “GP26–28” and “GP21–25” (*P* = .007) and (2) IgA, “GP11–20” and “GP0–10” (*P* = .031).

## 4 Discussion

After adjusting for confounding cohort and demographic effects, this study demonstrates that robust protein analytes measured by the MAP-RBM platform can be identified in CSF. Seven analytes (CD40A, FABP, HGF, Lp(a), PRL, RETN, and VEGF [Table 2]) showed significant correlations with CSF Aβ_1–42_ levels in the combined cohort. Four of them (Lp(a), PRL, RETN, and VEGF) showed consistent direction of associations across all individual cohorts as indicated by the effect sizes. We also found that VEGF was most significantly associated with MMSE in the combined cohort, followed by PPP, Lp(a), IgA, and TF.

We did not include CN subjects with abnormal CSF Aβ_1–42_ levels in the analysis as our goal was to identify MAP-RBM analytes that correlate with amyloid pathology. However, it is still of our interest to explore the characteristics of analytes in Table 2 for this group. We included back the 171 samples in this current analyses. Two analytes, PRL and VEGF remained significant (FDR *P* < .05) and had smaller effect sizes (same direction) across cohorts, suggesting the identified biomarkers were comparatively less effective in the nonsymptomatic population.

The identified top 10 analytes from our analysis are associated with different aspects of AD physiopathology. Lp(a), PRL, IgA, and TF have never been reported in previous individual studies using the CSF MAP-RBM panel [14], [15], [16].

CD40A is responsible for regulating immune response and is widely expressed in the brain [33]. It is also involved in microglial activation and brain inflammation in AD [34]. High baseline CD40A levels predicted reduced Aβ_1–42_ levels over time in ADNI [16], but this analyte was not reported in the other two studies [14], [15]. We also observed high CD40A levels in subjects with high Aβ_1–42_ levels (Supplementary Fig. 1B).

FABP may contribute to neurodegeneration via intracranial lipid metabolism [35]. It has been studied in CSF and serum in AD [36] and was also reported from both UPenn and WUSTL studies [14], [15]. The reported higher FABP levels in dementia subjects, as well as in early phases of AD [37], are consistent with our findings that FABP levels are higher in AD subjects in all cohorts (Supplementary Fig. 1A).

HGF is a potent mitogen for mature hepatocytes. It is expressed in astrocytes and is associated with white matter changes [38]. In WUSTL, AD subjects had slightly higher HGF levels in CSF as compared with CN [15]. Interestingly, we found HGF was correlated with Aβ_1–42_ levels in ADNI but not in WUSTL. This maybe because CN subjects with abnormal CSF Aβ_1–42_ levels were excluded in our analysis (Supplementary Fig. 1E).

Lp(a) protein (equivalent to LPA gene) consists of an low-density lipoprotein-like particle. Studies showed that increased plasma concentration of Lp(a) was associated with cerebrovascular disease [39]. Besides, evidence suggests that serum Lp(a) levels were highly correlated with the severity of AD [40], in line with what was observed in our cohorts (Supplementary Fig. 2B). However, it was not reported in any of the previous CSF–based MAP-RBM studies for diagnosis [14], [15], [16].

PRL is secreted by the pituitary gland and its elevated concentration in serum correlates with abnormalities in immune response [41]. The physiological importance of PRL is not fully known, but some suggested it maybe a regulator of stress response [42]. Same as Lp(a), PRL was not reported previously [14], [15], [16]. Its level was slightly higher in subjects with low Aβ_1–42_ levels than the others (Supplementary Fig. 1C).

RETN is a hormone likely associated with inflammation [43] and atherosclerosis [44]. It was a reported AD diagnostic marker in UPenn [14], and similarly, we observed higher levels of RETN in subjects with lower Aβ_1–42_ levels (Fig. 1B).

VEGF regulates vessel formation, axonal growth, and neuronal loss [45]. Low plasma and CSF VEGF levels in AD have been reported by other studies [38], previously in WUSTL [15], and were observed in all our cohorts (Fig. 1A). Findings from a recent study, however, suggest that VEGF_189_ levels were higher in AD and were involved in cognitive impairment via a role in neuroprotection and neurogenesis [46]. The higher VEGF levels of AD maybe due to the measurement of VEGF possessing a different immunoglobulin-like domain.

In our study, we identified three proteins which may reflect cognitive changes in the AD spectrum. PPP was one of the diagnostic targets reported in previous studies in both CSF and plasma [15], [29]. Its levels were altered in plasma clinical MCI/AD populations [47]. In our combined cohort, we found CSF PPP levels to be statistically different between samples with questionable and mild cognitive problems, suggesting this maybe a possible target for AD staging.

The roles for the other two proteins (IgA and TF) in AD are relatively less investigated. IgA was shown to improve the integrity of the blood-brain barrier (BBB) in rats [48]. The protective function of IgA to prevent breakdown of the BBB could delay or prevent AD [49]. On the other hand, TF may contribute to the formation of senile plaques, but the mechanism is not clear [50].

To conclude, using CSF samples from three different cohorts, we were able to identify robust analytes measured from the MAP-RBM platform. Focusing on samples whose cognitive status was consistent with their amyloid status, seven analytes were found to be statistically correlated with CSF Aβ_1–42_ levels. These analytes contribute differently in AD pathophysiology, including inflammatory response, lipid metabolism, atherosclerosis, and insulin resistance. Moreover, VEGF was strongly associated with cognitive impairment as measured by MMSE scores, followed by PPP. Although IgA and TF are relatively unexplored, they may reflect cognitive changes in the symptomatic AD samples. All these promising analytes need to be validated in a better well-designed study to verify their clinical utility.

**Research in context**

1. Systematic review: We used a multianalyte platform by Rules Based Medicine to uncover new protein analytes that were suggestive of the presence or absence of amyloid pathology quantified by cerebrospinal fluid (CSF) Aβ_1–42_ levels (Aβ_1–42_ cutoff defined by Shaw et al. using Alzheimer's Disease Neuroimaging Initiative data) and study how these biomarkers correlate with cognitive decline on symptomatic Alzheimer's disease subjects from three independent cohorts.
2. Interpretation: After adjusting for confounding effects, seven protein analytes (CD40 antigen fatty acid binding protein, hepatocyte growth factor, lipoprotein a, prolactin, resistin, and vascular endothelial growth factor [VEGF]) were highly correlated with abnormal CSF Aβ_1–42_ levels. VEGF, pancreatic polypeptide immunoglobulin A, and tissue factor / thromboplastin were associated with cognitive impairment as measured by mini-mental state examination.
3. Future directions: Only some of the identified analytes are known to be associated with AD physiopathology. In the future, we will include mild cognitive impairment subjects in our study. Further investigation is also required to study the longitudinal aspects of these analytes on subjects with different rates of cognitive decline.

## Figures and Tables

**Table 1:** Demographic of subjects included in CSF MAP-RBM from all three cohorts in our study (after removing subjects with disagreement of Aβ_1–42_ levels and clinical diagnosis labels)

Demographic	ADNI		UPenn		WUSTL		Combined	
Diagnosis	AD	CN	AD	CN	AD	CN	AD	CN
n (subjects)	64	58	225	22	56	73	345	153
Gender (F%)	45	52	59	68	63	62	57	58
Age, y (SD)	74 (8)	75 (5)	72 (9)	70 (11)	77 (7)	70 (7)	73 (8)	72 (8)
*APOE* ε4 alleles, %								
0	48	59	35	77	27	85	36	74
1	36	33	51	23	63	15	50	23
2	16	8	14	0	10	0	14	3
MMSE score at baseline (SD)	23 (2)	29 (1)	21 (6)	29 (1)	24 (3)	29 (1)	22 (5)	29 (1)
MMSE groups, %								
0–10	0	0	5	0	0	0	3	0
11–20	6	0	27	0	18	0	21	0
21–25	78	1	35	0	45	0	45	1
26–28	16	16	22	14	33	27	23	21
29–30	0	83	2	86	4	73	2	78

Abbreviations: CSF, cerebrospinal fluid; MAP-RBM, multianalyte platform by Rules Based Medicine; Aβ_1–42_, amyloid β; ADNI, Alzheimer's Disease Neuroimaging Initiative; UPenn, University of Pennsylvania; WUSTL, Washington University in St. Louis; AD, Alzheimer's disease; CN, cognitive normal; MMSE, mini-mental state examination score; SD, standard deviation.

**Table 2:** Analytes significantly associated with CSF Aβ_1–42_ levels (FDR adjusted *P* < .05) from univariate linear regression analyses in each individual cohort and in the combined cohort

MAP-RBM analyte	ADNI	UPenn	WUSTL	Combined
VEGF	0.067 (−0.56)	**0.009 (−0.46)**	**0.000 (−0.91)**	**0.000 (−0.48)**
FABP	**0.007 (0.79)**	**0.013 (0.98)**	0.554 (−0.03)	**0.000 (0.43)**
RETN	0.771 (0.34)	**0.000 (0.55)**	0.860 (0.22)	**0.000 (0.28)**
CD40A	0.106 (−0.49)	0.119 (0.13)	0.118 (−0.37)	**0.000 (−0.19)**
PRL	0.115 (0.42)	0.119 (0.46)	0.935 (0.23)	**0.005 (0.27)**
Lp(a)	0.739 (0.20)	0.051 (0.43)	0.735 (0.08)	**0.012 (0.18)**
HGF	0.023 (0.55)	0.286 (0.55)	0.935 (−0.01)	**0.013 (0.29)**

Abbreviations: CSF, cerebrospinal fluid; Aβ_1–42_, amyloid β; FDR, false discovery rate; MAP-RBM, multianalyte platform by Rules Based Medicine; ADNI, Alzheimer's Disease Neuroimaging Initiative; UPenn, University of Pennsylvania; WUSTL, Washington University in St. Louis; VEGF, vascular endothelial growth factor; FABP, fatty acid binding protein; RETN, resistin; CD40A, CD40 antigen; PRL, prolactin; Lp(a), lipoprotein a; HGF, hepatocyte growth factor.

NOTE. Effect sizes were shown in parenthesis. Bold text indicates *P* < .05.

**Table 3:** FDR adjusted *P* values obtained from ANOVA models on comparing means of analytes across different MMSE groups

	ADNI	UPenn	WUSTL	Combined
VEGF	0.994	0.003	0.244	0.001
PPP	0.994	0.014	0.244	0.004
Lp(a)	0.994	0.014	0.874	0.016
IgA	0.994	0.044	0.244	0.017
TF	0.994	0.014	0.988	0.044

Abbreviations: FDR, false discovery rate; ANOVA, analysis of variance; MMSE, mini-mental state examination; ADNI, Alzheimer's Disease Neuroimaging Initiative; UPenn, University of Pennsylvania; WUSTL, Washington University in St. Louis; VEGF, vascular endothelial growth factor; IgA, immunoglobulin A; LPA, lipoprotein a.
