# Supplementary material for: Identifying amyloid pathology–related cerebrospinal fluid biomarkers for Alzheimer's disease in a multicohort study
Source: Alzheimers Dement (Amst). 2015 Aug 1;1(3):339–48. doi: 10.1016/j.dadm.2015.06.008 (PMC4672388; doi:10.1016/j.dadm.2015.06.008)
Supplement: Supplementary Tables 1-4 and Supplementary Figs. 1 and 2 [file mmc1.docx]

**Supplementary Information**

**Tables**

**Supplementary Table 1:**  **Demographic of subjects included in CSF multi-analyte profiling from the three cohorts in our study**

|  | | ***ADNI*** | | ***UPenn*** | | ***WUSTL*** | | ***Combined*** | |
| --- | --- | --- | --- | --- | --- | --- | --- | --- | --- |
| **Diagnosis** | | ***AD*** | **CN** | ***AD*** | **CN** | ***AD*** | **CN** | ***AD*** | **CN** |
| **N (subjects)** | | 69 | 91 | 249 | 26 | 59 | 208 | 377 | 325 |
| **Gender (% F)** | | 43% | 49% | 57% | 62% | 61% | 66% | 55% | 60% |
| **Age, y (SD)** | | 75(8) | 76(6) | 72(9) | 70(11) | 76(7) | 70(7) | 73(9) | 72(8) |
| **ApoE E4 alleles** | 0 | 49% | 60% | 36% | 73% | 31% | 67% | 38% | 66% |
|  | 1 | 36% | 32% | 50% | 27% | 61% | 30% | 49% | 30% |
|  | 2 | 14% | 8% | 14% | 0% | 8% | 2% | 13% | 4% |
| **MMSE score at baseline (SD)** | | 24(2) | 29(1) | 21(6) | 29(1) | 25(4) | 29(1) | 22(5) | 29(1) |
| **MMSE groups** | 0-10 | 0% | 0% | 5% | 0% | 0% | 0% | 3% | 0% |
|  | 11-20 | 6% | 0% | 26% | 0% | 15% | 0% | 21% | 0% |
|  | 21-25 | 77% | 1% | 34% | 0% | 39% | 2% | 43% | 1% |
|  | 26-29 | 17% | 58% | 25% | 38% | 37% | 54% | 25% | 54% |
|  | 30 | 0% | 41% | 0% | 46% | 9% | 44% | 2% | 43% |

Abbreviations: AD = Alzheimer’s disease; CN = Cognitive Normal; MMSE = Mini-Mental State Examination score

**Supplementary Table 2:**  **52 MAP-RBM analytes that are in common across all three cohorts.**

| **Protein Name** | **Abbreviation** |
| --- | --- |
| Alpha1Antitrypsin | AAT |
| Angiotensin Converting Enzyme | ACE |
| Alpha2Macroglobulin | A2M |
| Angiopoietin 2 | ANG2 |
| Apolipoprotein AI | APOAI |
| Apolipoprotein CIII | APOCIII |
| Apolipoprotein H | APOH |
| Apolipoprotein a | Lp(a) / LPA |
| AXL Receptor Tyrosine Kinase | AXL |
| Beta 2 Microglobulin | B2M |
| Complement C3 | C3 |
| Calcitonin | CAL |
| CD40 antigen | CD40A |
| Chromogranin A | CGA |
| C Reactive Protein | CRP |
| Chemokine CC4 | HCC4 |
| Fatty Acid Binding Protein heart | FABP |
| Ferritin | FRTN |
| Fibrinogen | FIBRIN |
| Follicle Stimulating Hormone | FSH |
| Heparin Binding EGF Like Growth Factor | HBEGF |
| Hepatocyte Growth Factor | HGF |
| Intercellular Adhesion Molecule 1 | ICAM1 |
| Immunoglobulin A | IGA |
| Insulin like Growth Factor Binding Protein | IGFBP |
| Interleukin 16 | IL16 |
| Interleukin 8 | IL8 |
| Leptin | LEPTIN |
| Monocyte Chemotactic Protein 1 | MCP1 |
| Macrophage Migration Inhibitory Factor | MMIF |
| Macrophage Inflammatory Protein 1 beta | MIP1B |
| Matrix Metalloproteinase 3 | MMP3 |
| Myoglobin | MYO |
| Plasminogen Activator Inhibitor 1 | PAI-1 |
| Pancreatic Polypeptide | PPP |
| Prostatic Acid Phosphatase | PAP |
| Pregnancy Associated Plasma Protein A | PAPPA |
| Prolactin | PRL |
| T Cell Specific Protein | RANTES |
| Resistin | RETN |
| S100 calcium binding protein B | S100B |
| Serum Amyloid P Component | SAP |
| Stem Cell Factor | SCF |
| Serum Glutamic Oxaloacetic Transaminase | SGOT |
| Sex Hormone Binding Globulin | SHBG |
| Sortilin | SORT |
| Tissue Factor | TF |
| Tissue Inhibitor of Metalloproteinases 1 | TIMP1 |
| Tumor Necrosis Factor Receptor 2 | TNFR2 |
| TNF Related Apoptosis Inducing Ligand Receptor | TNFRAILR |
| Vascular Endothelial Growth Factor | VEGF |
| von Willebrand Factor | VWF |

**Supplementary Table 3: Effects of Age, gender and APOE on 52 MAP-RBM analytes across all three cohorts.** ANCOVA models were built and unadjusted p-values are listed. Those that surpass FDR adjusted p<0.05 are highlighted.

|  | ***ADNI*** | | | ***UPenn*** | | | ***WUSTL*** | | |
| --- | --- | --- | --- | --- | --- | --- | --- | --- | --- |
|  | **Age** | **Gender** | **APOE** | **Age** | **Gender** | **APOE** | **Age** | **Gender** | **APOE** |
| **AAT** | 0.057 | 0.031 | 0.530 | 0.004 | 0.058 | 1.000 | 0.021 | 0.006 | 0.240 |
| **ACE** | 0.077 | 0.330 | 0.290 | 0.640 | 0.420 | 0.320 | 0.310 | 0.390 | 0.024 |
| **A2M** | 0.016 | 0.690 | 0.740 | 0.000 | 0.023 | 0.990 | 0.000 | 0.640 | 0.370 |
| **ANG2** | 0.013 | 0.150 | 0.120 | 0.003 | 0.100 | 0.450 | 0.012 | 0.870 | 0.040 |
| **APOAI** | 0.160 | 0.170 | 0.810 | 0.061 | 0.750 | 0.170 | 0.091 | 0.009 | 0.024 |
| **APOCIII** | 0.030 | 0.210 | 0.100 | 0.026 | 0.510 | 0.560 | 0.001 | 0.037 | 0.690 |
| **APOH** | 0.019 | 0.000 | 0.800 | 0.005 | 0.005 | 0.470 | 0.005 | 0.000 | 0.930 |
| **LPA** | 0.430 | 0.096 | 0.150 | 0.150 | 0.340 | 0.035 | 0.340 | 0.003 | 0.850 |
| **AXL** | 0.260 | 0.710 | 0.160 | 0.720 | 0.650 | 0.210 | 0.850 | 0.990 | 0.041 |
| **B2M** | 0.002 | 0.190 | 0.470 | 0.000 | 0.510 | 0.790 | 0.016 | 0.660 | 0.093 |
| **C3** | 0.056 | 0.016 | 0.910 | 0.000 | 0.033 | 0.300 | 0.000 | 0.077 | 0.650 |
| **CAL** | 0.650 | 0.520 | 0.360 | 0.037 | 0.000 | 0.380 | 0.059 | 0.009 | 0.210 |
| **CD40A** | 0.000 | 0.370 | 0.014 | 0.001 | 0.065 | 0.520 | 0.051 | 0.029 | 0.180 |
| **CGA** | 0.290 | 0.380 | 0.550 | 0.690 | 0.065 | 0.310 | 0.840 | 0.270 | 0.140 |
| **CRP** | 0.640 | 0.350 | 0.830 | 0.008 | 0.200 | 0.000 | 0.170 | 0.400 | 0.000 |
| **HCC4** | 0.880 | 0.057 | 0.086 | 0.000 | 0.043 | 0.620 | 0.001 | 0.001 | 0.560 |
| **FABP** | 0.540 | 0.180 | 0.059 | 0.500 | 0.610 | 0.029 | 0.007 | 0.340 | 0.420 |
| **FRTN** | 0.010 | 0.087 | 0.420 | 0.001 | 0.008 | 0.420 | 0.060 | 0.320 | 0.250 |
| **FIBRIN** | 0.110 | 0.009 | 0.460 | 0.430 | 0.270 | 0.024 | 0.000 | 0.000 | 0.094 |
| **FSH** | 0.750 | 0.000 | 0.140 | 0.340 | 0.000 | 0.780 | 0.250 | 0.000 | 0.910 |
| **HBEGF** | 0.073 | 0.890 | 0.059 | 0.900 | 0.150 | 0.280 | 0.560 | 0.860 | 0.120 |
| **HGF** | 0.290 | 0.150 | 0.340 | 0.008 | 0.810 | 0.450 | 0.044 | 0.670 | 0.810 |
| **ICAM1** | 0.002 | 0.180 | 0.720 | 0.005 | 0.470 | 0.840 | 0.065 | 0.690 | 0.091 |
| **IGA** | 0.190 | 0.000 | 0.840 | 0.020 | 0.000 | 0.790 | 0.058 | 0.008 | 0.920 |
| **IGFBP** | 0.001 | 0.021 | 0.410 | 0.000 | 0.007 | 0.530 | 0.000 | 0.870 | 0.980 |
| **IL16** | 0.046 | 0.570 | 0.100 | 0.007 | 0.150 | 0.500 | 0.110 | 0.035 | 0.002 |
| **IL8** | 0.210 | 0.680 | 0.120 | 0.005 | 0.006 | 0.950 | 0.080 | 0.390 | 0.680 |
| **LEPTIN** | 0.510 | 0.000 | 0.460 | 0.023 | 0.000 | 0.260 | 0.034 | 0.000 | 0.550 |
| **MCP1** | 0.029 | 0.006 | 0.091 | 0.031 | 0.200 | 0.630 | 0.110 | 0.017 | 0.610 |
| **MMIF** | 0.350 | 0.850 | 0.500 | 0.012 | 0.460 | 0.570 | 0.000 | 0.470 | 0.038 |
| **MIP1B** | 0.310 | 0.860 | 0.580 | 0.220 | 0.410 | 0.940 | 0.350 | 0.660 | 0.570 |
| **MMP3** | 0.002 | 0.011 | 0.047 | 0.084 | 0.110 | 0.750 | 0.006 | 0.490 | 0.210 |
| **MYO** | 0.130 | 0.002 | 0.990 | 0.330 | 0.036 | 0.120 | 0.012 | 0.000 | 0.760 |
| **PAI-1** | 0.000 | 0.024 | 0.270 | 0.000 | 0.670 | 0.570 | 0.000 | 0.007 | 0.016 |
| **PPP** | 0.004 | 0.390 | 0.720 | 0.000 | 0.002 | 0.560 | 0.000 | 0.009 | 0.110 |
| **PAP** | 0.140 | 0.290 | 0.610 | 0.450 | 0.730 | 0.360 | 0.260 | 0.270 | 0.780 |
| **PAPPA** | 0.130 | 0.058 | 0.550 | 0.000 | 0.000 | 0.750 | 0.870 | 0.610 | 0.310 |
| **PRL** | 0.990 | 0.005 | 0.140 | 0.290 | 0.007 | 0.036 | 0.140 | 0.085 | 0.360 |
| **RANTES** | 0.950 | 0.730 | 0.500 | 0.030 | 0.470 | 0.490 | 0.014 | 0.490 | 0.710 |
| **RETN** | 0.680 | 0.480 | 0.024 | 0.000 | 0.580 | 0.260 | 0.000 | 0.220 | 0.620 |
| **S100B** | 0.110 | 0.190 | 0.430 | 0.031 | 0.042 | 0.140 | 0.040 | 0.027 | 0.440 |
| **SAP** | 0.140 | 0.000 | 0.110 | 0.210 | 0.000 | 0.950 | 0.310 | 0.000 | 0.170 |
| **SCF** | 0.620 | 0.590 | 0.100 | 0.000 | 0.380 | 0.350 | 0.100 | 0.930 | 0.005 |
| **SGOT** | 0.660 | 0.260 | 0.790 | 0.071 | 0.930 | 0.350 | 0.760 | 0.450 | 0.018 |
| **SHBG** | 0.007 | 0.160 | 0.710 | 0.000 | 0.009 | 0.470 | 0.000 | 0.600 | 0.990 |
| **SORT** | 0.440 | 0.920 | 0.500 | 0.210 | 0.350 | 0.330 | 0.220 | 0.710 | 0.980 |
| **TF** | 0.020 | 0.490 | 0.260 | 0.180 | 0.960 | 0.270 | 0.640 | 0.540 | 0.160 |
| **TIMP1** | 0.000 | 0.010 | 1.000 | 0.000 | 0.170 | 0.340 | 0.001 | 0.850 | 0.780 |
| **TNFR2** | 0.000 | 0.100 | 0.330 | 0.000 | 0.900 | 0.520 | 0.000 | 0.820 | 0.950 |
| **TNFRAILR** | 0.002 | 0.110 | 0.540 | 0.000 | 0.380 | 0.730 | 0.000 | 0.580 | 0.580 |
| **VEGF** | 0.003 | 0.330 | 0.790 | 0.064 | 0.990 | 0.520 | 0.950 | 0.620 | 0.004 |
| **VWF** | 0.000 | 0.460 | 0.220 | 0.000 | 0.160 | 0.810 | 0.000 | 0.680 | 0.970 |

**Supplementary Table 4: Adjusted p-values from the univariate linear regression analyses performed on each 54 MAP-RBM analytes in each of the cohorts (using CSF Aβ_1-42_ levels as outcome).** Highlighted are those that have surpass FDR adjusted p<0.05.

|  | ***ADNI*** | ***UPenn*** | ***WUSTL*** | ***Combined*** |
| --- | --- | --- | --- | --- |
| **AAT** | 0.866 | 0.992 | 0.935 | 0.945 |
| **ACE** | 0.942 | 0.286 | 0.118 | 0.129 |
| **A2M** | 0.968 | 0.235 | 0.897 | 0.466 |
| **ANG2** | 0.736 | 0.716 | 0.092 | 0.221 |
| **APOAI** | 0.739 | 0.286 | 0.712 | 0.829 |
| **APOCIII** | 0.739 | 0.794 | 0.935 | 0.954 |
| **APOH** | 0.771 | 0.178 | 0.935 | 0.390 |
| **LPA** | 0.739 | 0.050 | 0.735 | 0.012 |
| **AXL** | 0.417 | 0.510 | 0.554 | 0.134 |
| **B2M** | 0.866 | 0.968 | 0.118 | 0.619 |
| **C3** | 0.739 | 0.232 | 0.935 | 0.466 |
| **CAL** | 0.866 | 0.857 | 0.774 | 0.658 |
| **CD40A** | 0.106 | 0.119 | 0.118 | 0.001 |
| **CGA** | 0.942 | 0.794 | 0.239 | 0.492 |
| **CRP** | 0.106 | 0.178 | 0.935 | 0.864 |
| **HCC4** | 0.963 | 0.178 | 0.935 | 0.405 |
| **FABP** | 0.007 | 0.013 | 0.554 | 0.000 |
| **FRTN** | 0.739 | 0.704 | 0.935 | 0.466 |
| **FIBRIN** | 0.739 | 0.684 | 0.196 | 0.983 |
| **FSH** | 0.963 | 0.735 | 0.935 | 0.864 |
| **HBEGF** | 0.739 | 0.178 | 0.935 | 0.460 |
| **HGF** | 0.023 | 0.286 | 0.935 | 0.013 |
| **ICAM1** | 0.963 | 0.684 | 0.554 | 0.852 |
| **IGA** | 0.963 | 0.395 | 0.935 | 0.483 |
| **IGFBP** | 0.968 | 0.966 | 0.575 | 0.954 |
| **IL16** | 0.771 | 0.857 | 0.561 | 0.864 |
| **IL8** | 0.968 | 0.178 | 0.561 | 0.171 |
| **LEPTIN** | 0.968 | 0.945 | 0.712 | 0.864 |
| **MCP1** | 0.968 | 0.647 | 0.512 | 0.466 |
| **MMIF** | 0.106 | 0.671 | 0.935 | 0.102 |
| **MIP1B** | 0.973 | 0.286 | 0.935 | 0.492 |
| **MMP3** | 0.736 | 0.934 | 0.209 | 0.935 |
| **MYO** | 0.739 | 0.178 | 0.935 | 0.248 |
| **PAI-1** | 0.739 | 0.286 | 0.092 | 0.065 |
| **PPP** | 0.417 | 0.422 | 0.512 | 0.102 |
| **PAP** | 0.968 | 0.723 | 0.935 | 0.829 |
| **PAPPA** | 0.955 | 0.684 | 0.963 | 0.974 |
| **PRL** | 0.115 | 0.119 | 0.935 | 0.005 |
| **RANTES** | 0.968 | 0.178 | 0.554 | 0.102 |
| **RETN** | 0.771 | 0.000 | 0.860 | 0.000 |
| **S100B** | 0.700 | 0.966 | 0.935 | 0.829 |
| **SAP** | 0.771 | 0.253 | 0.935 | 0.470 |
| **SCF** | 0.963 | 0.713 | 0.092 | 0.974 |
| **SGOT** | 0.968 | 0.647 | 0.935 | 0.864 |
| **SHBG** | 0.942 | 0.857 | 0.935 | 0.829 |
| **SORT** | 0.247 | 0.338 | 0.935 | 0.936 |
| **TF** | 0.963 | 0.713 | 0.092 | 0.487 |
| **TIMP1** | 0.963 | 0.330 | 0.935 | 0.466 |
| **TNFR2** | 0.771 | 0.178 | 0.712 | 0.221 |
| **TNFRAILR** | 0.968 | 0.981 | 0.935 | 0.954 |
| **VEGF** | 0.067 | 0.009 | 0.000 | 0.000 |
| **VWF** | 0.739 | 0.713 | 0.897 | 0.956 |

**Figures**

**
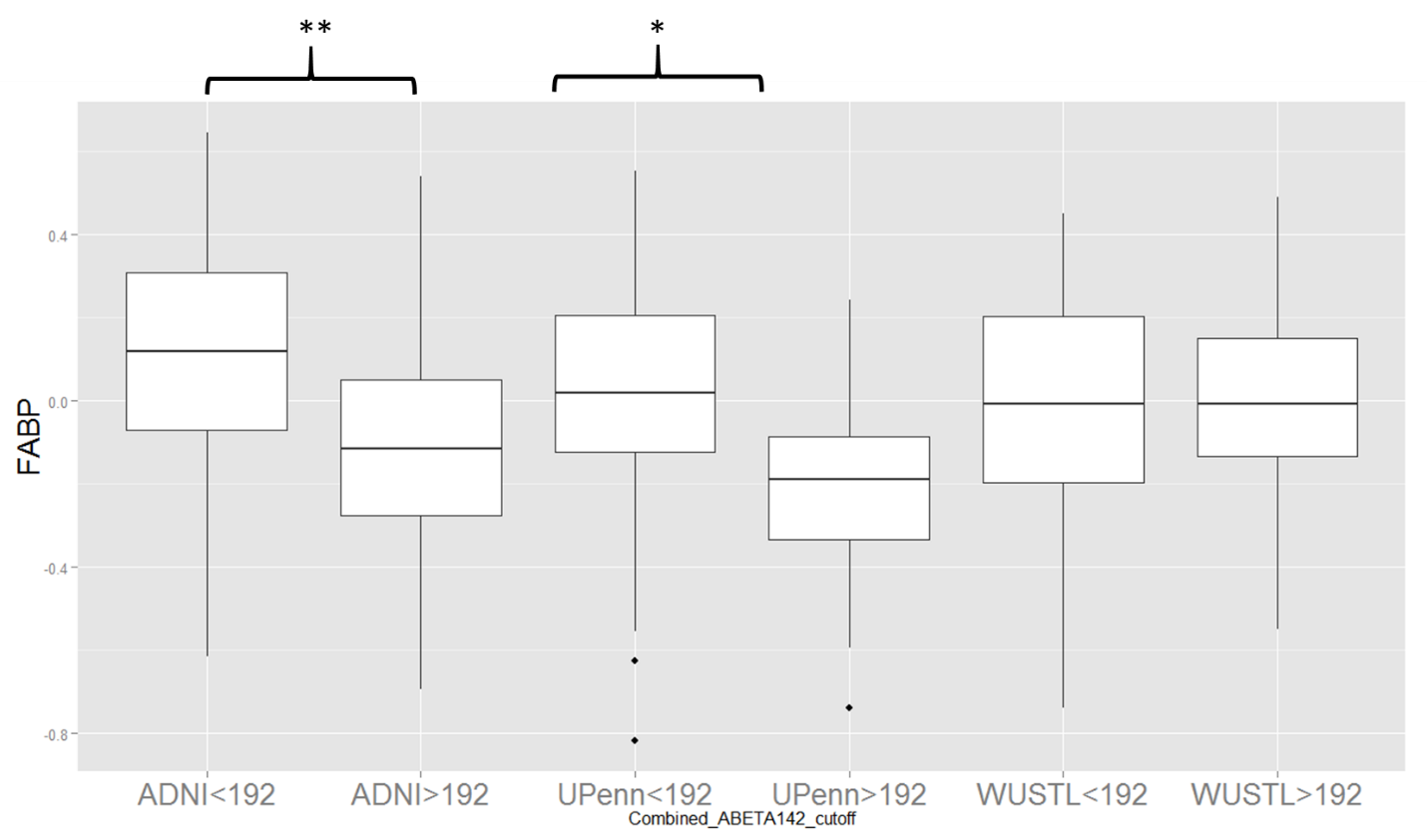
**

1. **FABP**


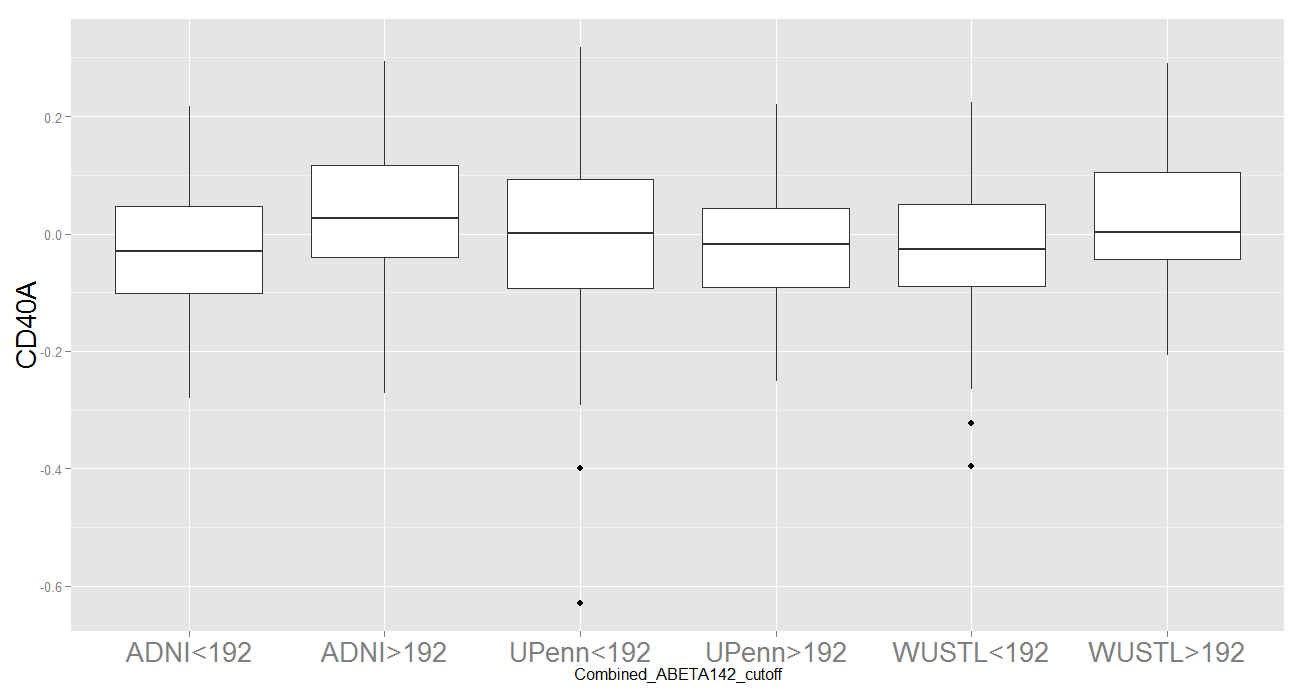


1. **CD40A**


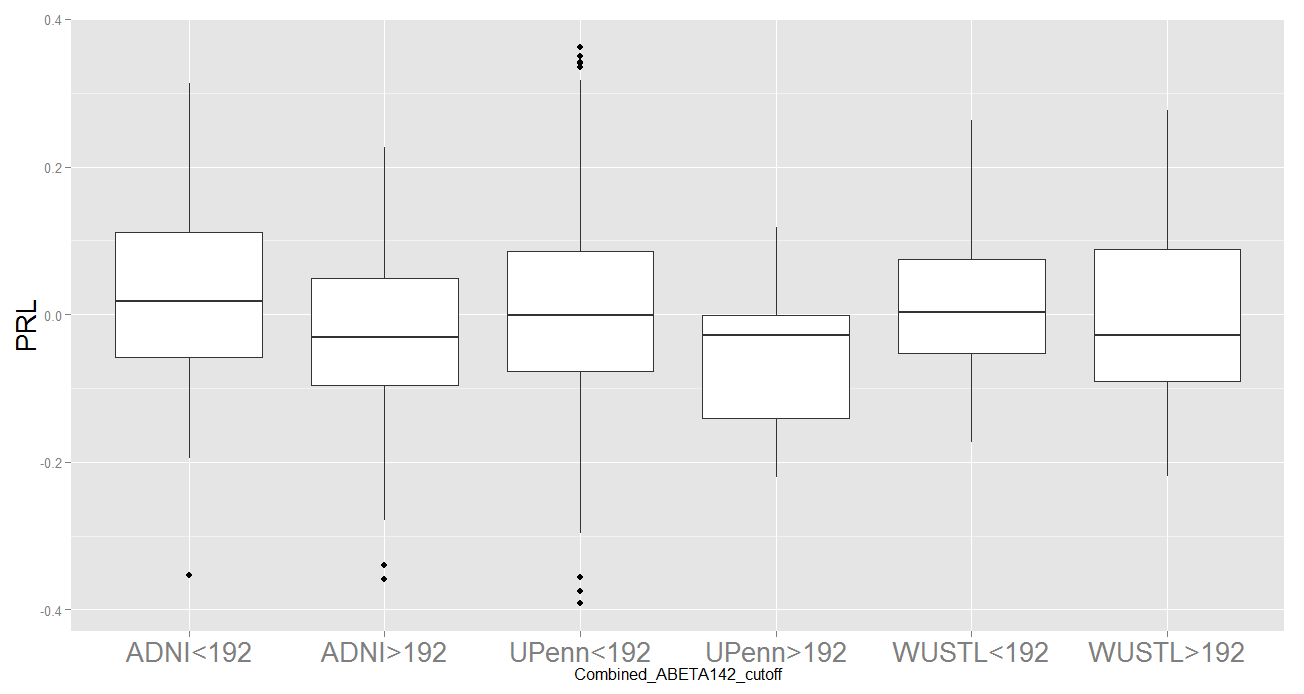


1. **PRL**

**
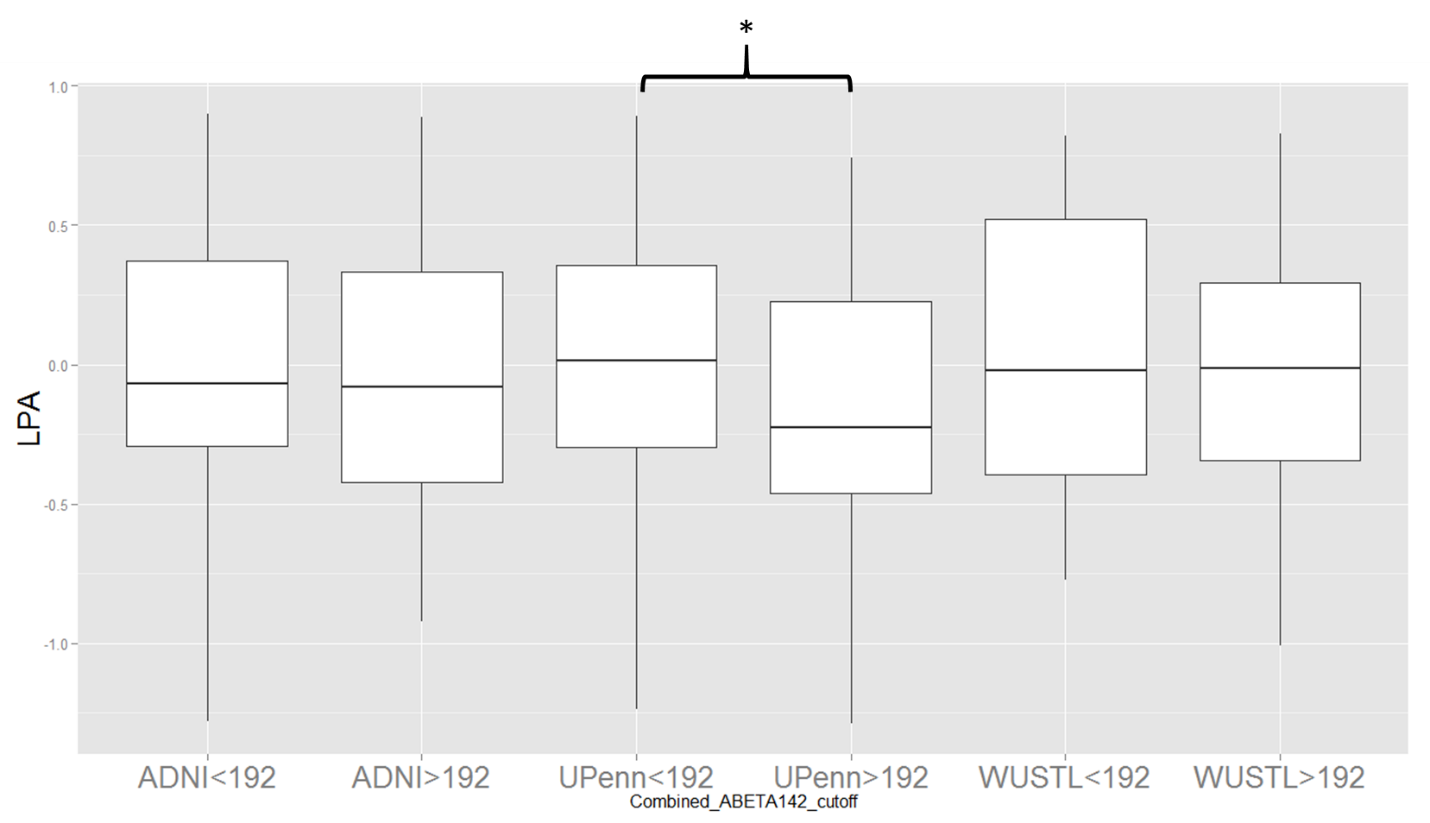
**

1. **LPA**

**
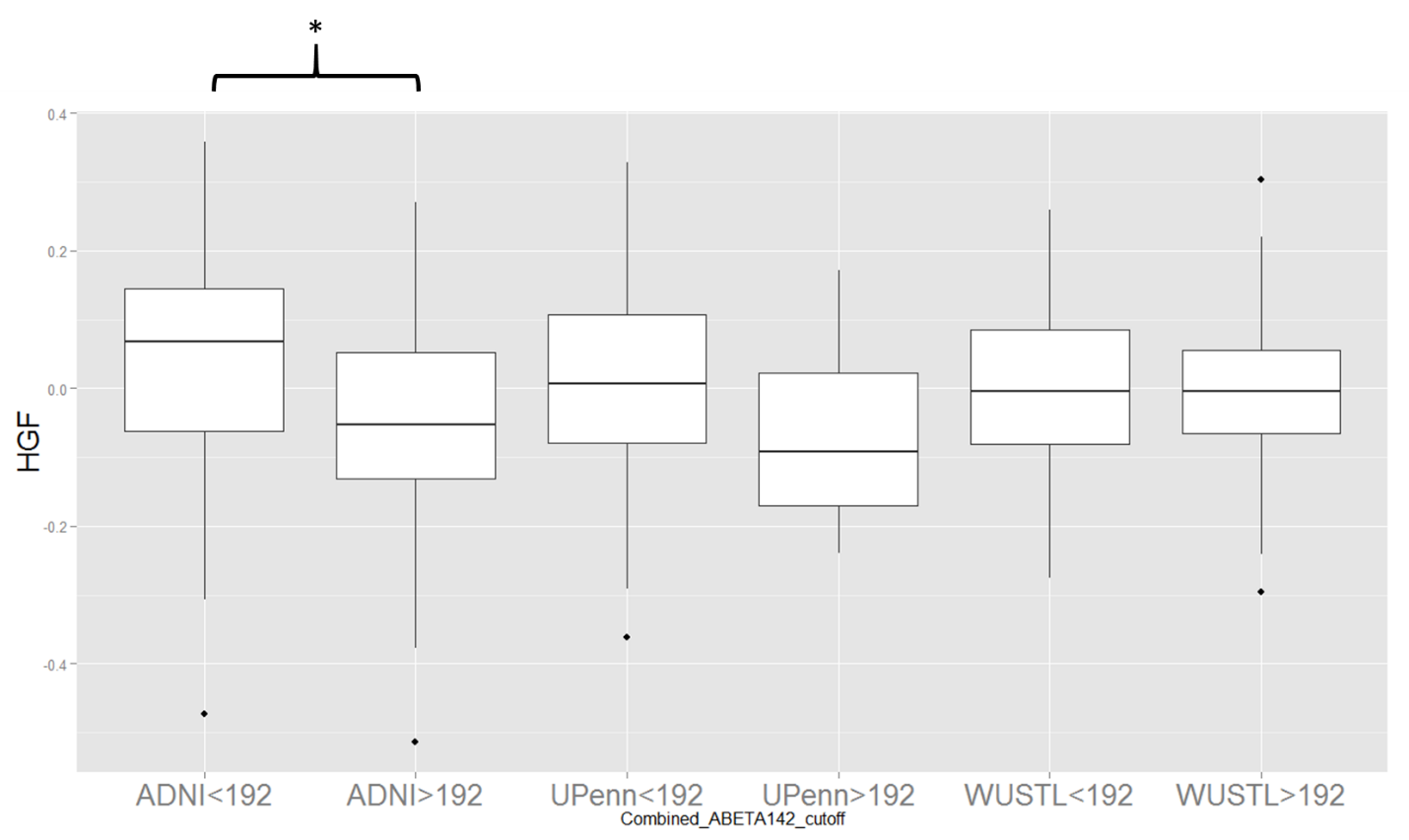
**

1. **HGF**

**Supplementary Figure 1: MAP-RBM analytes that significantly associated with Aβ_1-42_ levels in some of the cohorts. a) FABP; b) CD40A; c) PRL; d) LPA; and e) HGF.**


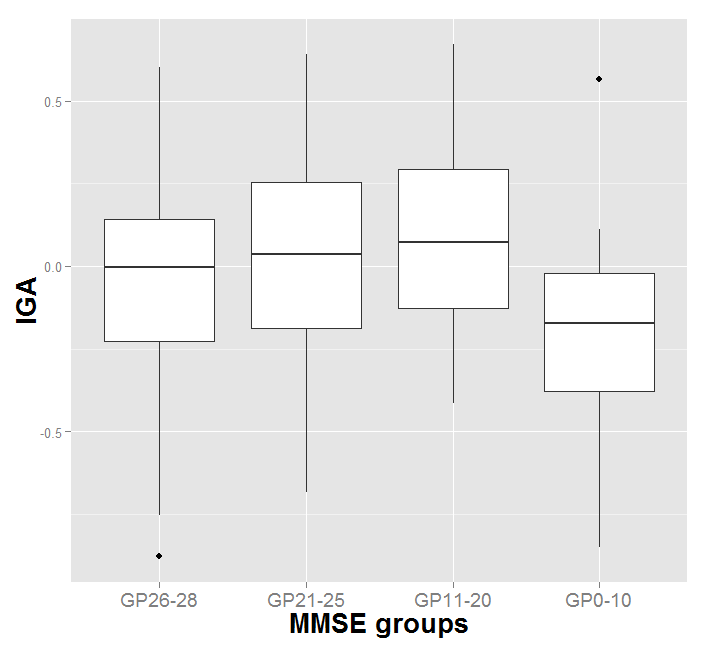


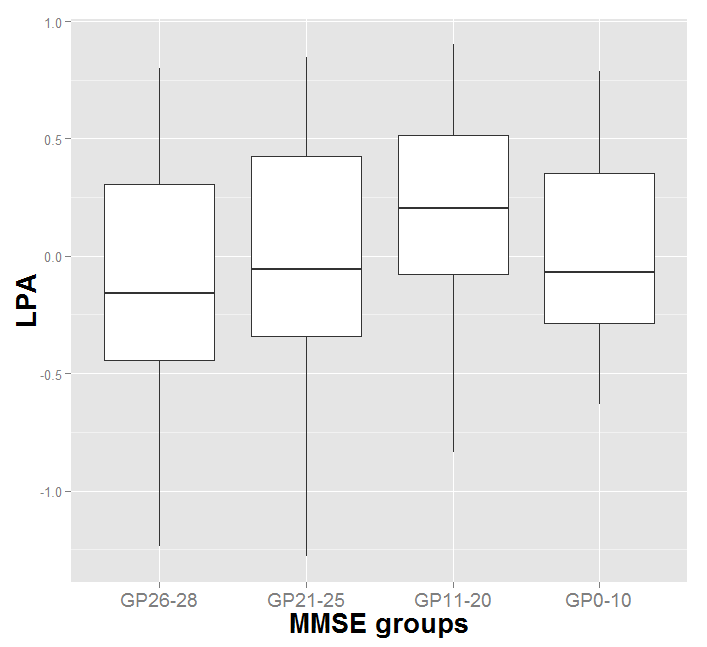


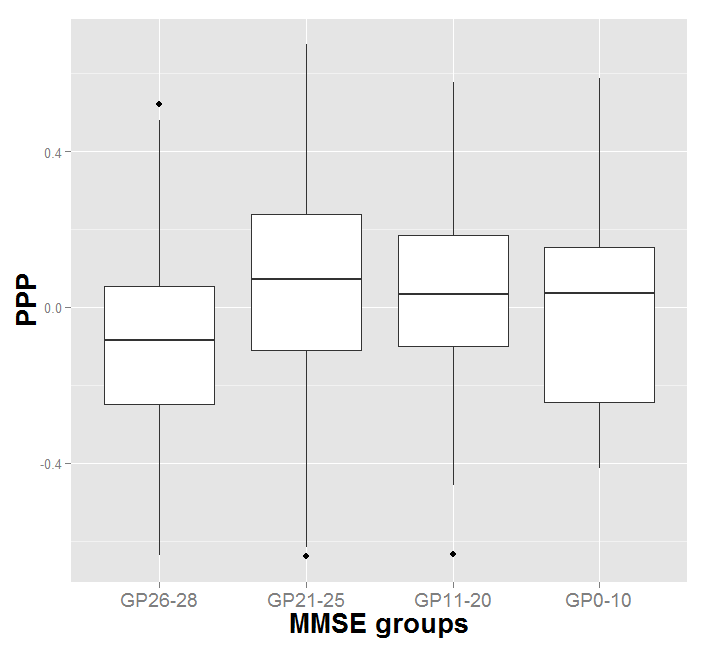


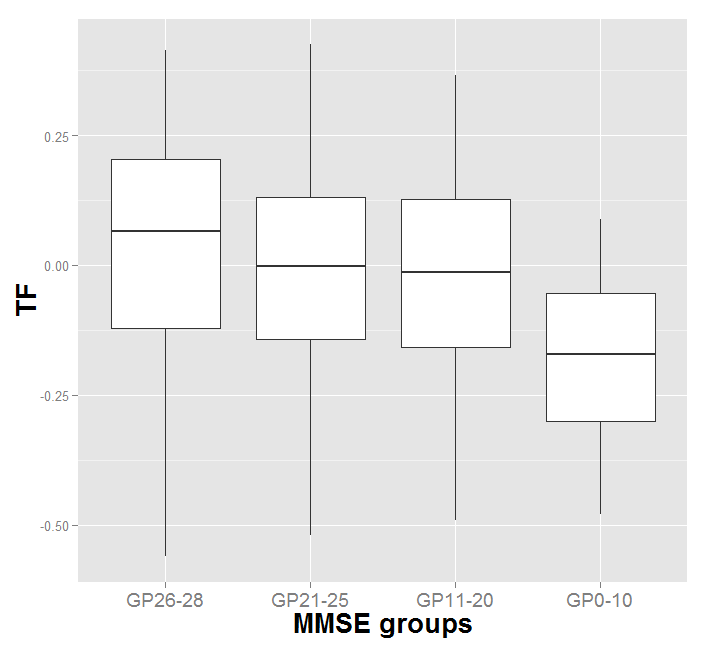


**Supplementary Figure 2: MAP-RBM analytes across MMSE groups in the combined cohorts: a) IGA; b) LPA; c) PPP; and d) TF.**
